# Basic Life Support Training Methods for Health Science Students: A Systematic Review

**DOI:** 10.3390/ijerph16050768

**Published:** 2019-03-03

**Authors:** Mario García-Suárez, Carlos Méndez-Martínez, Santiago Martínez-Isasi, Juan Gómez-Salgado, Daniel Fernández-García

**Affiliations:** 1 University Hospital of León, 24008 León, Spain; mgarcs06@estudiantes.unileon.es (M.G.-S.); cmendm00@estudiantes.unileon.es (C.M.-M.); 2 Health and Podiatry Unit, Department of Health Sciences, Faculty of Nursing and Podiatry, Universidade da Coruña, Campus de Esteiro, 15403 Ferrol, Spain; santiago.martinez.isasi@udc.es; 3 Department of Nursing, University of Huelva, 21007 Huelva, Spain; 4 Safety and Health Posgrade Program, Espíritu Santo University, Samborondón, 092301 Guayaquil, Ecuador; 5 Department of Nursing and Physiotherapy, University of Leon, 24071 León, Spain; dferg@unileon.es

**Keywords:** training, health students, cardiorespiratory resuscitation, basic life support

## Abstract

The acquisition of competencies in basic life support (BLS) among university students of health sciences requires specific and updated training; therefore, the aim of this review was to identify, evaluate, and synthesise the available scientific knowledge on the effect of training in cardiorespiratory resuscitation in this population. A comprehensive literature search was conducted in MEDLINE, CUIDEN, Web of Science, Wiley Online Library, CINAHL, and Cochrane, including all randomised clinical trials published in the last ten years that evaluated basic life support training methods among these students. We selected a total of 11 randomissed clinical trials that met the inclusion criteria. Participants were nursing and medicine students who received theoretical and practical training in basic life support. The studies showed a great heterogeneity in training methods and evaluators, as did the feedback devices used in the practical evaluations and in the measurement of quality of cardiorespiratory resuscitation. In spite of the variety of information resulting from the training methods in basic life support, we conclude that mannequins with voice-guided feedback proved to be more effective than the other resources analysed for learning.

## 1. Introduction

Cardiorespiratory arrest (CRA) has become a major public health problem and one of the leading causes of death in the Western world in recent years. Cardiopulmonary resuscitation (CPR) is the technique used in the cases of CRA. It consists of thoracic compressions (which are important for the perfusion of vital organs) and rescue breaths by means of artificial ventilation [1,2,3,4,5,6,7]. The quality of CPR is vitally important, and it depends on the level of knowledge and skills held by those who carry out the CPR. Even among healthcare professionals, that level can be inadequate. Therefore, an improvement in educating healthcare professionals in CPR techniques may increase survival rates in cases of CRA [2,8,9].

Within a hospital, the nursing staff is usually the first group of professionals to identify CPR, so competence in basic life support (BLS) is a key factor in recognising cardiac arrest, activating emergency systems, initiating effective CPR, and safely using the defibrillator [10,11,12,13]. Roh and Issenberg concluded that technical skills in CPR among nursing students are very poor, and that despite efforts to improve the quality of psychomotor skills, the results obtained are still not encouraging [5].

As has previously been described, BLS is a fundamental therapy for saving lives, and it requires a broad knowledge of cognitive and psychomotor skills. [13,14] In spite of this, several studies have shown that BLS education is difficult: learners’ retention of motor skills is poor (even immediately after they have completed the course), causing less-than-ideal performance of CPR [6,14,15]. In addition, if those who have been trained in CPR do not frequently perform it, their skills deteriorate over a period of between 3 and 6 months. Therefore, it is very important that in addition to developing different learning strategies, these should be combined with other recycling (retraining) measures during that period of time [10,16].

Within CPR teaching, different methods have been proposed, such as simulation, classical instructor-led teaching, and self-directed mannequins with continuous verbal feedback, which have been shown to be much more effective for retaining knowledge and motor skills [9,12,17,18]. Other methods of learning may be based on interactive videos, high-fidelity 3D simulation scenarios, and partner-based training, in which very positive results have been obtained [1,19].

With all this, there is a need for a systematic review that includes a comparison in the methods used (traditional versus alternative) trying to find the most effective for the teaching of BLS, CPR, and use of automatic external defibrillators (AEDs) in university health science students.

Finally, the research question selected by the authors was what is the most effective method for teaching of BLS, CPR techniques, and use of AED for health science students?

So that, the main objective of this systematic review was to identify, evaluate and synthesize what kind of method is more effective of training in basic life support, cardiopulmonary resuscitation techniques and use of automatic external defibrillator among health science students.

## 2. Materials and Methods

We undertook a systematic review in line with the Preferred Reporting Items for Systematic Reviews and Meta-Analyses (PRISMA) statement [20].

### 2.1. Literature Search

The search for articles was conducted during February and March 2017. The scientific databases searched were MEDLINE, CUIDEN, Web of Science, Wiley Online Library, CINAHL, and Cochrane.

We used both English and Spanish descriptors that were located in the Medical Subject Headings (MeSH) and in *Descriptores de Ciencias de la Salud* (Health Sciences Descriptors; DeCS). These included “health science”, “students”, “cardiopulmonary resuscitation”, “training”, “traditional”, “new methods”, “motor skills”, “simulation”, and “evaluation of efficacy-effectiveness”. Descriptors that were synonymous with one another were combined in the search with the Boolean “OR” operator, while the “AND” operator was used to interrelate different concepts.

As an example, one of the search strategies used in the Medline database was: “health science students” AND (“traditional cardiopulmonary resuscitation training” OR “new methods cardiopulmonary resuscitation training”).

### 2.2. Eligibility Criteria

The studies that were selected for the systematic review met the following inclusion criteria.

- Year of publication: we included all articles published between 2007 and 2017, in order to obtain the most recent articles on training methods.
- Language: Spanish and/or English.
- Studies: we included full texts of randomised clinical trials (RCTs), because these epidemiological studies provide more evidence.
- Population: students of both sexes who were pursuing university degrees related to the health sciences.
- Intervention: any method used in the teaching of BLS and the acquisition of technical skills in CPR in adults.
- Results: we selected studies that contained information about the socio-demographic characteristics of participants, ones that analysed the effect of training in the acquisition of theoretical and practical knowledge, and ones that reported on measurement tools for skills relating to placement of the hands, number of compressions, average depth of compressions, number of ventilations, or volumes administered.

All articles that did not meet these criteria were excluded.

### 2.3. Selection of Articles and Data Extraction

Initially, two reviewers independently performed the article search in order to minimise selection bias. After deleting duplicates, an initial selection of articles was carried out following independent analysis of the titles.

We then conducted a second review that included the reading of titles, abstracts and key words of the articles found by the two reviewers, who jointly proceeded to make the final selection of articles. At this point, a third independent reviewer intervened in the decision-making process in cases of disagreement. Finally, after obtaining the full-text articles, the third and final selection took place, in which the articles that were eventually used in the review were chosen.

Because the studies reviewed were very heterogeneous and had different methods of intervention and assessment, it was not possible to undertake a meta-analysis.

Last of all, and following the final selection of the articles included in the review, we extracted the following information from each article: first author, publication year, population, study groups, learning method, evaluation method, immediate results, and results after refreshers/recycling.

### 2.4. Evaluation of the Studies’ Methodological Quality

The methodological quality of the randomised clinical trials included in the review was evaluated using the Jadad scale [21].

The validity of this scale has been proven in the scientific literature, and it is simple and quick to use. In addition, the researchers were already trained in its use, having deployed it in other studies.

The scale gives a score between 0 and 5 points, primarily to three aspects: randomisation, blinding (double-blind), and description of withdrawals and dropouts during follow-up. A score of 5 represents the highest possible methodological quality, while a score of under 3 means that the evaluated clinical trial is of a low methodological quality. Below, we provide the full scale with the items and their corresponding scores (Table 1).

## 3. Results

### 3.1. Study Characteristics and Quality Evaluation

Through our search, we obtained a total of 522 articles that were potentially eligible for the review. Of them, 371 were eliminated on the basis that they were duplicates from across the different databases.

After completing the first selection (reading of titles), 109 articles were excluded. We then analysed the abstracts of the 42 articles that were still potentially valid for inclusion, through which a total of 18 were excluded. Finally, and after obtaining the remaining 24 articles in full-text form, a total of 11 studies were included in the review [6,9,11,12,13,16,17,18,19,22,23].

**Figure 1:**
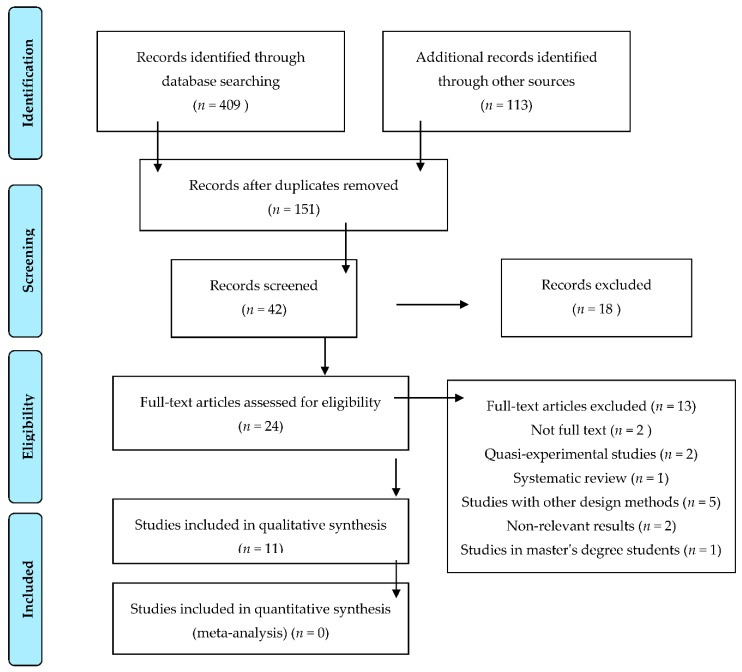
Flowchart with selection of articles included in the review.

The excluded articles were those which did not meet the inclusion criteria for the study, which are shown in a flow diagram in Figure 1.

Table 2 presents the final articles that were part of the systematic review based on their methodological quality.

The scores obtained on the Jadad scale for the analysed articles ranged from 2 to 5 points, with an average of 2.81 points. Only one article with double blinding [22] obtained the maximum score, and four articles [9,12,19,23] (which were transversal and involved only one measure) scored 2 points. All the studies, with the exception of the one conducted by Isbye et al. [22], presented a high risk of bias, as they involved single blinding, making it impossible for there to be double-blinding for participants and researchers.

All the data extracted from each article, with general and specific characteristics, are summarised in Table 3.

### 3.2. Study Participants

The participants of the different studies were university students from different branches of the health sciences, including mainly nursing students [11,12,13,17,18,19] and medicine students [22,23]. Only one study did not distinguish the students’ degree titles [6].

The analysed studies included a total of 2175 participants; that by Partiprajak et al. (*n* = 30) [13] had the fewest participants, while that by Oermann et al. (*n* = 606) [19] had the most.

### 3.3. Participants’ Knowledge Prior to the Undertaking of the Studies

Information about previous knowledge of and technical skills in BLS and CPR was collected in seven articles [9,11,12,13,16,18,22]. This meant that some authors excluded a certain number of participants from studies [9,16], or conversely used this information as a basis when establishing prior training [11,12,13,18,22].

### 3.4. Teaching Methods and Duration

The different teaching methods primarily related to two aspects: theoretical content aspects, and aspects derived from the acquisition of technical skills in CPR. To this end, the researchers were guided by the recommendations of the American Heart Association (AHA) [9,12,13,17,18,23] and of the European Resuscitation Council (ERC) [6,11,19,22]. The exception in this regard was the study by Mpotos et al. [16], which does not mention any recommendations. These guides were also employed later to carry out measurements in the evaluations.

For the theoretical level of the training, the authors employed different techniques according to the different groups that they created within their studies. Accordingly, instructors taught lectures using visual media such as presentations or videos [6,9,11,12,17,23]. In other cases, the participants acquired knowledge independently through computer CDs or DVDs provided by the researchers [9,13] or through different tests with self-assessments [16,19]. In the rest of the studies, training methods were not specified [18,22].

As for the teaching of skills, the most used method was traditional instructor-led teaching, which appeared in a total of 6 studies [9,11,12,17,22,23]. In the study by Li et al. [23], one of the group’s first underwent a pre-assessment (a practical scenario), which after being recorded and reviewed by the instructors, was subsequently used for training purposes as feedback.

The second-most-used method was training with mannequins that had feedback systems (these are known as mannequins with a skill reporter). These featured in five studies [6,11,13,16,19]. In addition, in four studies the participants from some groups carried out self-directed learning with mannequins that, in addition to feedback, had voice prompts that corrected errors (the so-called voice advisory mannequin, VAM) [9,12,18,22]. It is also worth mentioning that in the studies by Aqel et al. [17] and Boada et al. [19] high-fidelity simulation programs were used to deliver the training.

Finally, in two studies [6,9], the control groups did skills training without any kind of feedback or supervision from instructors, and in two others [16,18] no skills practice of any kind was performed.

In terms of the duration of the training undertaken to acquire knowledge and skills, the most homogeneous approach was the traditional teaching method’s time frame of between four and five hours, except in the case of the study carried out by Spooner et al. [6], which took place over 8 h. There were large variations in the other studies.

### 3.5. Methods Used in the Evaluation

Evaluation methods were organised according to knowledge and skills. To measure knowledge levels, the methods used were pre-intervention [23], post-intervention [9,19] or pre- and post-intervention [11,13,17] questionnaires, or a subjective assessment by instructors of performance in the sequence of BLS steps [6,17]. In addition, two studies included a questionnaire about the confidence that participants had in executing the skills after the training [11,13]. The measurement of CPR-technique skills during the period in which they were being acquired was taken in 10 of the 11 studies through a skill reporter mannequin [6,9,11,12,13,16,18,19,22,23].

### 3.6. Results Obtained after the Intervention

The different results obtained after analysing the articles were divided into two groups for drafting purposes. We will first discuss the results of the studies in which a single measure was used following completion of the intervention, and we will then consider the other studies, in which more evaluations were carried out over time.

Studies that involved a measurement that evaluated theoretical knowledge reported an improvement in all groups [19,23], with the exception of the study by Roppolo et al. [9], in which the control group that received theoretical training with an instructor obtained better results. In the study by Kardong Edgren et al., knowledge was not measured [12].

As for the acquisition of technical CPR skills, the groups that acquired knowledge through a VAM obtained better results with statistically significant differences relative to the rest of the groups [9,12]. In the study that used high-fidelity simulation, no differences between the groups were established [19]. Finally, in the study by Li et al., in which a group carried out a practical scenario with feedback provided by instructors to participants, significant results were obtained in CPR technique for all aspects except for the placement of hands (both groups obtained 100%) [23].

On the other hand, in the first assessment of the studies that applied several measurements [11,13], knowledge improved in all groups, with the exception of the study by Aqel et al. [17], in which the improvement was additionally statistically significant in the intervention group. In the study by Spooner et al. [6], the correct completion of the BLS algorithm was evaluated, with no differences between the groups. In addition, the studies by Hernández Padilla et al. [11] and by Partiprajak et al. [13] used questionnaires to analyse the confidence of participants in performing the CPR technique safely, and they noted an improvement in results after the intervention had been completed.

With respect to skills in performing CPR, in three studies [11,13,16], no differences were found between the different groups, while in the studies by Spooner et al. and Aqel et al. [6,17], the intervention groups performed better in both studies. Finally, in the study by Isbye et al. [22], the instructor-led group obtained better results than the self-directed groups that used a VAM.

### 3.7. Results Obtained after a Retention Period or Refreshers

Seven studies carried out a subsequent measurement after a refresher or simply by applying a knowledge retention period [6,11,13,16,17,18,22] over periods of time ranging from 6 weeks [6] to 1 year [18] after the intervention.

In six studies, a measurement for knowledge retention was used [6,11,13,16,17,22]. In the study by Spooner et al., after 6 weeks the group that had undertaken practice obtained better results, while there were no differences when it came to correctly applying the BLS algorithm. [6] In the study by Hernández Padilla et al., the results were better for the self-directed group at three months. [11] In the study by Partiprajak et al., after three months, worse results were obtained in terms of knowledge, similar results were found in terms of confidence of participants when performing CPR, and better results were obtained in terms of acquisition of CPR skills [13]. The study by Mpotos et al. observed that the control group that had not received practical skills training obtained better results than the intervention group at 6 months [16]. In the fifth study in which retention of knowledge was evaluated, carried out by Aqel et al. [17], it was observed that after 3 months the improvement in results for knowledge and skills in the group that received the high-fidelity simulation remained. Finally, the study by Isbye et al. concluded that there were no differences between the groups after 3 months [22].

In the study by Oermann et al. [18] there was no measurement of skills immediately after the intervention. Out of their control and intervention groups, they produced random subgroups at 3, 6, 9, and 12 months, which were the ones evaluated. Among these groups, no differences were established in terms of the number of compressions, volume: minute, or hand placements, but there were in relation to depth and volume administered, which decreased significantly as the measurements were taken over time. Finally, a fifth subgroup that was given a refresher was also established at 12 months, and statistically significant differences between the control and intervention groups were not obtained within it.

## 4. Discussion

To conduct this review, we drew on a total of 11 randomised clinical trials that were found in different databases and that aimed to assess the quality of training in CPR and BLS knowledge and technical skills among health sciences students. Most of the studies were conducted among nursing and medicine students, in line with the study by López Messa et al., which highlights that BLS training for future healthcare professionals should be reinforced at the undergraduate level, especially in nursing and medicine degrees [24].

The studies included in the review were of a low methodological quality according to the Jadad scale. In view of these findings, one priority that emerges is the need to increase the number of RCTs with methodological rigour, which would make it possible to minimise biases and facilitate the identification of progress in scientific evidence regarding BLS training among health sciences students. To this end, the use of this same scale in other reviews or similar studies would facilitate this process.

The articles are also characterised by the absence of homogeneity in establishing BLS training, technical CPR skills and use of AEDs. Despite this, the results have shown how studies that used VAM [9,11,12,18] improved all skills immediately or in the long term, though in the study by Isbye et al. [22] ventilations were not improved at first.

Moreover, the realisation of practical cases through different simulation programs of high fidelity provided better results fundamentally in the acquisition of theoretical knowledge [17,19].

Weidman et al. define learning through simulation as an essential part of training, whether it is high or low fidelity [25]. High-fidelity simulation is very useful when comparing the results obtained with real outcomes, even though it requires thorough intervention from the instructors [26,27]. In addition, this training provides realistic environments and is more student focused [28].

The use of a skill reporter or VAM mannequins with feedback results in a remarkable rise in the improvement of the quality of CPR performed by nursing and medicine students, since it allows them to correct their mistakes or undertake knowledge refreshers independently, making it feasible to not have an instructor on an ongoing basis. Along this line, the study by Nielsen et al. concludes that this type of learning improves knowledge and skills [29].

Finally, in the studies included in the review, the use of AED is scarcely mentioned. Although eight studies included AEDs as part of the theoretical and practical training [6,9,11,13,17,19,22,23], only Roppolo et al. [9] implemented a measure concerning the use of this device. They obtained unfavourable results that do not coincide with those of the study by Ahn et al. [30], the main finding of which was that students reduced intervention times as soon as they had an AED nearby. It has been shown that courses of between 2 and 4 h in the use of an AED may be enough to operate them safely [31].

Therefore, and despite the fact that the use of AEDs is a priority when it comes to saving lives, there is a need for more studies that more comprehensively evaluate training in and handling and application of these devices in order for there to be fuller performance within BLS.

### Limitations

One of the main limitations of the study is that in spite of BLS and CPR training for health science students, the number of randomised clinical trials is not very high, and studies that have appeared are very heterogeneous in terms of how they have been produced. Moreover, after reviewing the studies on a methodological level, we observed that it is necessary to increase their methodological rigour.

Another of the limitations of the study is the fact that the recommendations issued by the AHA and ERC for BLS training evolve continuously, meaning that the inclusion of studies published over the last 10 years makes it very difficult to assess them in the same way.

In relation to the use of AEDs, it was not possible to describe them because most of the selected studies did not include measurement results.

Finally, researchers have not included students taking different degrees in their studies, so it has been impossible to establish differences between students, their degrees, and different training methods that it may have been possible to use.

## 5. Conclusions

The studies included in this systematic review are characterised by a low methodological quality and heterogeneity in terms of their interventions.

Findings have shown that the use of VAMs was more effective for learning CPR skills than the other resources analysed. With regard to the knowledge acquired, participants did not show differences between those who received a theoretical session with an instructor and participants who acquired knowledge independently through computer CDs or DVDs.

Studies did not show results of the use of AEDs, so a comparison could not be made. Therefore, we would recommend future researchers to include in their research the use of AED, since we consider it necessary to increase information regarding its use and how students can face its use in a real case. Finally, we would recommend that future research have a high methodological quality so that studies can have greater relevance.

## Figures and Tables

**Table 1:** Jadad scale.

	Criterion	Scores
**1**	Was the study described as random?	Yes: 1 point No: 0 point
**2**	Was the randomisation scheme described and appropriate?	Yes: 1 point No: 0 points
**3**	Was there a description of dropouts and withdrawals?	Yes: 1 point No: 0 points
**4**	Was the randomisation scheme described and appropriate?	Yes: 1 point No: −1 point
**5**	Was the study described as double-blind?	Yes: 1 point No: −1 point

**Table 2:** Methodological quality of studies, calculated with the Jadad scale. BLS basic life support; AED: automatic external defibrillator.

Article	Jadad Scale Items					Jadad Score
	1	2	3	4	5	
*An evaluation of objective feedback in basic life support (BLS) training* [6]	1	0	1	1	-	3
*A randomised controlled trial comparing traditional training in cardiopulmonary resuscitation (CPR) to self-directed CPR learning in first year medical students: The two-person CPR study* [9]	1	0	0	1	-	2
*Effects of two retraining strategies on nursing students’ acquisition and retention of BLS/AED skills: A cluster randomised trial* [11]	1	0	1	1	-	3
*Comparison of two instructional modalities for nursing student CPR skill acquisition* [12]	1	0	0	1	-	2
*Retention of basic life support knowledge, self-efficacy and chest compression performance in Thai undergraduate nursing students* [13]	1	0	1	1	-	3
*Repetitive sessions of formative self-testing to refresh CPR skills: A randomised non-inferiority trial* [16]	1	0	1	1	-	3
*High-fidelity simulation effects on CPR knowledge, skills, acquisition, and retention in nursing students* [17]	1	0	1	1	-	3
*Effects of monthly practice on nursing students’ CPR psychomotor skill performance* [18]	1	0	1	1	-	3
*Using a serious game to complement CPR instruction in a nurse faculty* [19]	1	0	0	1	-	2
*Voice advisory manikin versus instructor facilitated training in cardiopulmonary resuscitation* [22]	1	1	1	1	1	5
*Pre-training evaluation and feedback improve medical students’ skills in basic life support* [23]	1	0	0	1	-	2

**Table 3:** Description of the studies included in the review. VAM: voice advisory mannequin.

Article	Population (Sample)	Study Groups and Teaching Methods	Assessment Methods	Immediate Results	Results after Refresher/Retention Period
*An evaluation of objective feedback in basic life support (BLS) training* [6]	Birmingham health centre students (*n* = 100)	Teaching in BLS and AED (8 h). Subsequently two groups with loss of two participants. (1) Control group: CPR practice without feedback. (2) Intervention group: CPR practice with a skill reporter	Sequence checklist evaluated by instructors and mannequin with a skill reporter. Use of AED not evaluated	Statistically significant differences since participants with practice with skill reporter obtained better averages in depth and % of correct compressions and better administered air volumes. There were no differences in terms of execution of the algorithm.	At 6 weeks, percentage of correct compressions was higher in participants who practised with a skill reporter. Volume administered increased in both groups. There were no differences in terms of execution of the algorithm.
*A randomized controlled trial comparing traditional training in cardiopulmonary resuscitation (CPR) to self-directed CPR learning in first year medical students: The two-person CPR study* [9]	First-year medical students (*n* = 180); neligible = 240. Those who had done BLS training in the past 5 years were excluded	(1) Self-directed learning with VAM for 2 h. (2) Normal mannequins and theory on a DVD for free viewing and practice for 2.5 h.Groups 1 and 2 could practise 10 days prior to the measurement. (3) Traditional group with instructor for 4–5 h on the day of assessment.	Simulations checklist evaluated by instructors and mannequins with a skill reporter	No statistically significant differences were established. Traditional group obtained better results for knowledge in simulations than the others. Main failure: misuse of AED. In terms of skills, there were significant differences only in the compressions: minute ratio, which was higher in the traditional group.	No
*Effects of two retraining strategies on nursing students’ acquisition and retention of BLS/AED skills: A cluster randomised trial* [11]	Nursing students based in Almería (Spain) and the United Kingdom (*n* = 177). Prior 3-h course, 3 months before	(1) Self-directed group: 4 h refresher in which required aspects were reviewed. (2) Instructor-led group: same time frame, but the instructor set goals and provided teaching in use of the material and final evaluation.	Pre- and post- intervention knowledge questionnaire, confidence questionnaires and mannequins with the skill reporter.	Both groups improved their skills, knowledge and confidence	At 3 months, there was a new assessment, in which skills and knowledge were improved, with the self-directed group obtaining better results.
*Comparison of two instructional modalities for nursing student CPR skill acquisition* [12]	Nursing students (*n* = 604) with prior BLS knowledge	(1) Mannequin-based group: theoretical class and training with mannequin (VAM). (2) Traditional instruction group: 4 h with instructor who taught knowledge and trained in skills with mannequin.	Mannequin with a skill reporter	The sample that undertook self-learning with mannequin obtained best results in all of the individual skills except frequency and compressions: ventilations ratio.	No
*Retention of basic life support knowledge, self-efficacy and chest compression performance in Thai undergraduate nursing students* [13]	Third-year university nursing students based in Thailand (*n* = 30), randomised with neligible = 180	One group, comprising women only. They had knowledge acquired 1 year previously. 1 h. BLS video followed by CPR practice with 1 and 2 resuscitators for 20 min.	Questionnaire on pre- and post- knowledge, questionnaire on confidence and mannequin with a skill reporter	No one passed the knowledge pre-test but 100% passed the post-test. In terms of confidence, an increase with the pre-test was noted. Motor skills were only recorded post-course, with 100% results for hand placement and decompression.	At 3 months, there was a new knowledge and skills test. Worse results were obtained for knowledge (30% passed), but there were better results in all skills compared to the other assessment (without refreshers). Values were maintained in terms of confidence.
*Repetitive sessions of formative self-testing to refresh CPR skills: A randomised non-inferiority trial* [16]	Third-year medical students based in Ghent (*n* = 218). People with knowledge excluded: nfinal = 196	After excluding from sample those with appropriate skills knowledge, a computer created two groups: (1) performed self-assessments in BLS training; (2) same training, and also practised CPR. Had 6 weeks to be proficient in CPR skills. Retention at 6 months	Mannequin with a skill reporter (2 min).	At the end of the first 6 weeks, there were no significant differences between the groups that were deemed proficient in CPR.	At 6 months, decrease in the number of people in both groups that performed quality CPR. Despite this, those who did not practice during the first 6 weeks obtained better results in depth and ventilations.
*High-fidelity simulation effects on CPR knowledge, skills, acquisition, and retention in nursing students* [17]	First-year nursing students (*n* = 90)	(1) Control Group: 4 h theory and traditional training with AED. (2) Intervention group: 4 h theory and training with high-fidelity simulation with AED.	Pre- and post- knowledge questionnaires and evaluation of skills by instructor during cardiac arrest activity.	Significant differences in the skills and knowledge among groups, with improvements in the intervention group.	New measurement at 3 months, in which knowledge and skills remained better in the intervention group.
*Effects of monthly practice on nursing students’ CPR psychomotor skill performance* [18]	Nursing students from different universities in U.S. neligible = 727; nfinal = 606	All students were trained in BLS at their universities. After this: (1) Control group: no practice; (2) Intervention group: CPR skills practice, 6 min a month, with VAM. At 3, 6, 9, and 12 months a random subgroup of each main group underwent measurement. Another subgroup, 12R, was also created, which was given a BLS refresher and subsequent measurement at 12 months.	Mannequin with a skill reporter	Throughout the study, there were no differences found in the compressions: minute ratio, hands placement and volume: minute. There were differences in depth and volume administered, with decreases in the control group.	In the 12R groups, which had a refresher, there were no differences, since both groups received a refresher in CPR knowledge and skills.
*Using a serious game to complement CPR instruction in a nurse faculty* [19]	Nursing students based in Norway (*n* = 109)	(1) Three control groups (A, B, C). Pre-test and practice with simulation with mannequins and with AED. (2) Five LIfe Support Simulation Activities (LISSA-2) groups (D, E, F, G, H). Tutorial focused on serious game simulation program with problems to be solved before the intervention.	Checklist questionnaires and mannequins with a skill reporter	Improved knowledge among those belonging to LISSA groups. In terms of skills, there were no differences between the groups. They conclude that this is a good method to support theory but that it does not improve skills.	No
*Voice advisory manikin versus instructor facilitated training in cardiopulmonary resuscitation* [22]	Medical students based in Copenhagen (*n* = 43). Students undertook course 1 year before.	(1) Instructor-led group: received teaching in skills for 32 min. (2) Mannequin-based group: use of a VAM for 5 min	Measurement of skills through a skill reporter, pre and post, 2 min	In the post-measurement, the instructor-led group obtained better results for skills.	At 3 months, new 2-min measurement, in which no differences between groups were found.
*Pre-training evaluation and feedback improve medical students’ skills in basic life support* [23]	Third-year medical students based in Sichuan (China) (*n* = 40)	(1) Control group: 45 min theory class followed by 45 min of traditional training with mannequin; (2) Intervention group: same theory class, followed by pre-assessment after simulation with instructor feedback for 15 min, followed by 30 min training with mannequin.	Questionnaires on prior knowledge and mannequin with a skill reporter	There were no differences upon analysis of theoretical knowledge. After evaluation with a skill reporter, better results with significant differences in the intervention group, except in hand positioning, which was the same.	No
